# A contentious intervention to support the medical workforce: a case study of the policy of introducing physician associates in the United Kingdom

**DOI:** 10.1186/s12960-024-00966-1

**Published:** 2025-01-17

**Authors:** Martin McKee, Louella K. Vaughan, Giuliano Russo

**Affiliations:** 1 London School of Hygiene & Tropical Medicine, 15-17 Tavistock Place, London, WC1H 9SH UK; 2 Barts Health NHS Trust, The Royal London Hospital, London, E1 1FR UK; 3 Wolfson Institute of Population Health, Queen Mary University of London, Charterhouse Square, London, EC1M 6BQ UK

## Abstract

**Background:**

Health systems across Europe are facing a workforce crisis, with some experiencing severe shortages of doctors. In response, many are exploring greater task-sharing, across established professions, such as doctors, nurses, and pharmacists, with patients and carers, and with new occupational groups, in particular ones that can assist doctors and relieve their workload.

**Case presentation:**

In the early 2000s the United Kingdom created a new occupational role, that of physician assistant. They had a science degree and then underwent a 2-year postgraduate training course. The name soon changed, to physician associate, and the range of roles and responsibilities expanded greatly, although in a largely unregulated manner; by 2024, some were undertaking complex procedures or managing undifferentiated patients in primary care. Catalysed by some high-profile failings, this expansion has generated major concerns, over patient safety and consent, the scope of practice and preferential employment conditions of this group, the adverse consequences for medical training, and the additional medical workload involved in supervision. This has led to a widespread grassroots backlash by the medical profession, often challenging their leaders who had supported this idea. As a consequence, professional bodies that were initially in favour are now expressing serious concerns and it seems likely that the roles and responsibilities of physician associates (and related occupations) will be curtailed. We review published literature and official documentation about this policy to understand the drivers of its development, its benefits, and risks.

**Conclusions:**

The experience in the UK offers cautionary lessons for other European countries contemplating similar ideas. It underscores the importance of maintaining trust with those affected by change, undertaking a detailed systems analysis with attention to risks of unintended consequences, agreeing clear role definitions, providing adequate regulatory oversight, and the need to avoid damaging training of future doctors. This case study highlights the need for a carefully thought-out approach that considers both the potential benefits and pitfalls of integrating new roles like physician associates into a healthcare system. The failure to do so has created a new occupational group with unrealistic expectations and has further demoralised an already unhappy medical profession.

## Introduction

Almost all countries face a healthcare workforce crisis, with those in Europe no exception [1]. It is estimated that the world will face a shortfall of 10 million health workers by 2030 [2]. The problems are greatest with the health professions, especially doctors.

Training in medicine has always been long. Diagnosis and treatment of all but the simplest of ailments require a detailed knowledge of the structures of the body, how it works, how it can go wrong, and how to put it right. To this must be added communication skills and understanding of why some groups of people are healthier than others [3]. The cognitive manipulation of the vast body of knowledge that underpins a consultation, termed ‘clinical reasoning’, must be explicitly taught and cannot be decoupled from the clinical context [4]. Once qualified, doctors must undertake 5–8 years further training to become a consultant or general practitioner.

The length and complexity of medical training and the risk that doctors must bear has meant that they have been amongst the most highly respected and well remunerated members of society. However, those who might have seen medicine as an attractive career now have many other options. This has coincided with increased pressure on health budgets in many countries, with medical incomes falling behind. Budgetary pressures have also, in some countries, led to deteriorating working conditions. Consequently, many health systems, even if not yet experiencing a crisis of recruitment, are facing one of retention [5].

One response has been to consider whether roles and responsibilities can be assumed by others (or, increasingly, by technology). There is now considerable literature on such task-sharing, much involving the transfer of roles and responsibilities among different types of health worker but also from health workers to patients or carers [6]. This typically show that while tasks that are relatively straightforward, such as some technical procedures or managing uncomplicated chronic diseases within protocols, can be transferred safely and efficiently to those with less training (presumably at lower cost), this is not the case for more complex activities, especially where advanced clinical reasoning skills are needed.

This debate generally overlooks a crucial point. There is a vast difference between transferring roles and responsibilities from one profession to another, such as medicine, nursing, and pharmacy, and transfers to occupations, such as community health workers or clinical assistants, who must be supervised and who lack the features of a profession such as a distinct body of complex knowledge [7].

Intended to inform responses to the unfolding medical workforce crisis in Europe [8, 9], this case study reports a policy in which this distinction was overlooked, the creation of the physician associate (PA) in the United Kingdom (UK). We review how this role evolved, the concerns that have arisen, and the responses it has elicited. We conclude by extracting lessons to be learned from this policy experience for other European countries contemplating something similar. The methods we employed and how we addressed reflexivity are described in Box 1.

## The emergence of an idea

The concept of a health worker that can take on some of the responsibilities of a doctor is not new, being a common response to severe shortages of doctors in, for example, isolated rural areas (such as feldshers in Russia [10] or officiers de santé in France. They re-emerged as Physician Assistants in the United States in the 1960s to address severe doctor shortages in rural areas [11]. This title made clear that their role was to assist a doctor under whose supervision they worked. In time, some conducted certain technical procedures, such as endoscopies or minor surgical procedures but they were not substitutes for doctors (or nurses) [12].

In the early 2000s, this concept was advocated as a partial solution to the UK’s problems of increasing costs of an ageing population and a looming medical workforce crisis. Some US-trained physician assistants were recruited [13]. The UK Association of Physician Assistants (UKAPA) was created in 2005 and the Royal Colleges of General Practitioners and of Physicians developed a curriculum in 2006. The first university courses were launched in 2008. Recruits required an undergraduate degree in science and their training, a mix of traditional and problem-based learning, lasted 2 years. It is commonly described as following a “medical model”, although that is never defined. A voluntary register was created, requiring those registered to undertake continuing professional development and be recertified every 6 years. In 2013, the approximately 300 UKAPA members voted to change the name of their occupation from ‘physician assistant’ to ‘physician associate’. The UKAPA was absorbed into the Royal College of Physicians (RCP), creating the Faculty of Physician Associates (RCPFPA) [14].

The long-term ambition was to introduce statutory regulation, allowing them to become prescribers and order ionising radiation; the registration body was initially intended to be the Health and Care Professionals Council (HCPC), which regulates 15 professions with legally protected titles, such as occupational therapists. However, lobbying by the PAs and some Royal Colleges succeeded in moving this to the General Medical Council (GMC), which regulates doctors. A 2024 statutory instrument enacted this [15], a mechanism that does not require usual levels of parliamentary scrutiny. It came into effect at the end of 2024 and covers and cover physician and anaesthetic associates (PAs and AAs).

The academic literature on PAs in the UK highlights the complexity of identifying their specific contribution to service provision, the lack of regulation surrounding a new cadre, and the limitations of their roles [16, 17].

The number of PAs in the National Health Services (NHS) of the four countries of the United Kingdom was still low until 2021 (fewer than 1000), but this has increased rapidly. They are employed in various specialities, including general practice (GP). In that case, their advocates see them as a response to the situation whereby “general practice is at crisis point, with many GP posts unfilled and experienced nurses leaving the NHS” [18]. The NHS created a funding stream that provides financial support for general practices employing a range of professions and occupations, including PAs, but not initially, GPs, although that decision has been reversed following the election of a new government. However, there is a growing backlash from patients and doctors against any further expansion. In the next section, we ask why this is happening.

## The emergence of concerns

Concerns that have been raised about this new occupational group fall, broadly, into six categories, patient safety, scope of practice, informed consent, preferential employment conditions, additional workload, and impact on medical training. While we draw on accounts in media reports and professional and academic journals for this case study; for completeness we must note that much of the debate has taken place on social media, where a large amount of material, ranging from documents, such as job descriptions, and experiences of health professionals have been shared.

## Patient safety

This attracted widespread attention when a young woman, with a condition that confers an increased risk of blood clots, developed leg pain and shortness of breath. She was seen twice by someone she understood was a general practitioner but was a PA, was prescribed paracetamol and propranolol, even though PAs cannot legally prescribe, and the practice policy was that any return consultation after seeing a PA should be with a doctor. She subsequently died from a pulmonary embolism [19]. Since then there have been other examples in the media raising concerns about patient safety [20], including at least another three deaths. In one, a young man with aortic dissection died after being discharged from hospital. The coroner issued a Prevention of Future Deaths report, which said, “It is a matter of concern that despite the patient’s reported symptoms, in view of his age and extensive family history of cardiac problems [he] was discharged … without being examined or reviewed in person by a doctor” [21]. In another case, a PA undertook a cystoscopy on a patient who had signs of active infection and failed to document a need for antibiotics. The patient later died of sepsis [22]. In a third, a PA kept an abdominal drain in situ and clamped after it should have been removed. Again, the coroner’s Prevention of Future Deaths report raised concerns about the scope of practice and supervision of PAs [23]. Since then, several further examples have emerged but it is impossible to obtain a systematic assessment of the scale of the problem because of limitations in NHS data systems [24].

Other concerns relate to ordering of ionising radiation, and the prescribing of drugs (including opiates), both of which are illegal, with reports that it is common practice for PAs to pre-write a script that a GP will ‘sign off’ with minimal scrutiny [25]. Other concerns have arisen following revelations about PAs undertaking medicolegal child protection work, which was not in line with national recommendations and could have prevented prosecutions, a practice that was stopped once news became public [26].

## Scope of practice

Scope of practice for healthcare practitioners is usually determined by a combination of qualifications and training, various laws (including those pertaining to negligence) and shared understandings both intra-professionally and with the public about what the limits of good practice ought to be. Instead, the NHS has determined that employers should decide on scope of practice for PAs and AAs [27].

While PAs are supposed to be dependent practitioners, always supervised by a consultant or a general practitioner, PAs are routinely seeing undifferentiated patients (those who do not have a diagnosis or management plan) in many settings, including general practice and emergency departments. As the supervising doctor is accountable for decisions as to how much to delegate to the PA, this places them in jeopardy if things go wrong. Of more concern, job descriptions and clinical rotas demonstrate that PAs are substituting for doctors at every grade, including taking on tasks normally restricted to specialists. The most egregious examples include PAs being deployed on consultant stroke rotas. Another concern is that, in general practice, the introduction of PAs is having the paradoxical effect of freeing up GPs not for more complex cases but for the growing volume of administrative work [28].

## Informed consent

A survey of a representative sample of the public conducted by the British Medical Association (BMA) found that a quarter believed that a PA is a doctor and many thought they are more senior than junior doctors [29]. This raises important questions about whether patients can provide truly informed consent as to who they will be seen by.

## Preferential employment conditions

Despite having less training, newly qualified PAs in the NHS are paid substantially more than doctors at the same stage in their career. Resident doctors are required to move both jobs and hospitals, sometimes every 3 months, while PAs mostly remain in the same location. Also, unlike doctors, many PAs work normal working hours. At the time of writing, the starting salary for a PA is approximately 35% more than for a newly qualified doctor [30]. Given that these less well-paid doctors may be asked to take responsibility for decisions made by PAs, such as which medications to prescribe, the scope for demoralisation is obvious.

The damage to morale is exacerbated by reports of increasing numbers of unemployed GPs, including some being replaced by PAs [31]. Locum posts are becoming increasingly rare and there are reports of over 50 applicants for substantive jobs [32].

## Additional workload

The assumption is that PAs should make doctors more efficient. In the hospital setting, it was assumed that PAs would undertake more administrative and minor clinical tasks, such as taking blood samples, allowing resident doctors to focus on their training. In general practice, it was assumed that if other healthcare workers, including PAs, would see more straightforward cases, GPs would be freed to focus on the patients who were sicker and/or more complex.

These assumptions overlook several considerations. Firstly, there are other occupational groups, such as clinical assistants (staff with vocational qualifications who provide administrative support and perform basic clinical tasks) [33], phlebotomists, and ward clerks, who can reduce hospital doctors’ workloads. Secondly, measures to address the notoriously poor information technology infrastructure in the NHS would help. Third, several studies have now shown that, far from reducing workload, the time and energy required to supervise PAs and other non-medical staff is burdensome, with GPs dissatisfied and expected efficiency improvements and cost reductions often failing to materialise [34]. Their expansion in primary care owes much to the Additional Roles Reimbursement Scheme (ARRS) that, in effect, makes them free to general practices which would otherwise have employed doctors. Economic analyses of PAs in the USA [35] and AAs in the UK [36] have suggested that their utility in the hospital setting has been overestimated, to the point where they are ‘economically nonviable’.

Many arguments that introducing PAs and AAs is efficient hinge on the assumption that they can be supervised remotely by the named clinician. However, this has been challenged by a 2017 GMC ruling, where a medical registrar, deemed to be the supervising doctor, failed to physically examine a patient clerked by a PA, leading to the registrar’s suspension [37].

## Impact on medical training

In the United Kingdom, post-graduate medical training is highly structured, with junctures at which the doctor’s subsequent direction is determined. The first is the transition from medical school to foundation training. The number of foundation posts should match the number of graduates, but the gap has widened in recent years and, in 2022, 791 applicants (395 of whom had graduated from UK medical schools that year; the remainder either holdovers from previous years or from other countries) were placed on a reserve list. This was up from 45 in 2016 [38]. There is a concern that the increasing shortfall in the number of places is due to hospitals employing PAs, thereby reducing training capacity. This is exacerbated by reductions in medical training budgets, with the funds released being explicitly repurposed to pay for the expansion in the numbers of PAs [23]. Additionally, a new allocation system for foundation places has left many newly qualified doctors dissatisfied. These developments have further eroded the morale of a workforce already despondent about falling pay and poor working conditions [39].

For non-consultant doctors at all levels (foundation programme, training grades and specialty doctors), introduction of PAs has led to the loss of opportunities to acquire skills and experiences. Time in theatre and other procedural work is not just highly valued but necessary for trainees. In a survey by the Association of Surgeons in Training (ASIT), 70.5% of respondents reported a negative impact on surgical training [40]. In another survey of members of the Royal College of Physicians of London, 58% of respondents said that having PAs on their team limited training opportunities for doctors [41]. This clearly ignores the consequences for the pipeline of future specialists.

A third concern is the expansion of PA university courses and the corresponding demand for clinical placements when there is already a severe shortage of placements for medical students, a situation that is becoming critical due to the parallel, and poorly thought out expansion of medical schools [42].

## The reaction

The concept of physician (and anaesthesia) associates initially attracted widespread support from the medical Royal Colleges, organisations in the UK which play a major role in advancing standards of care. Some, such as the Royal College of Physicians of London, even agreed to host a newly created Faculty of Physician Associates. The number of PAs was quite small for many years, and most doctors had little or no contact with them. However, the growth in numbers, delays to and problems with legislation on regulation, and the release of a Long-Term Workforce Plan [43], which set out an ambition to expand both the numbers and scope of practice of the non-medical workforce, has led to questions of why medical leaders were so supportive of an idea now being revealed as problematic.

The first move came from a group of anaesthetists concerned with the safety implications of expanding the AA workforce and reducing training for anaesthetic trainees [44]. An Extraordinary General Meeting of the Royal College of Anaesthetists was called in October 2023, with 89% of the 5 000 virtually attending members voting for a pause in the recruitment of AAs [45].

About the same time a survey of members of the Royal College of Paediatrics and Child Health found that 60% did not believe that PAs supported service delivery and 86% (90% of trainees) disagreed that they supported training [46]. In the survey by the ASIT mentioned above, 46.8% said that PAs had a negative impact on patient care [40].

The Extraordinary General Meeting of the Royal College of Physicians was marred by the misleading presentation of survey data that wrongly suggested support for PAs, leading to resignations of senior College officials [47] (in fact, nearly 72% felt there was a negative impact on training opportunities) [47]. The meeting passed resolutions calling for a pause in expansion of PAs and clarity on their scope of practice while calling for a “limit to the roll-out of the PA role” [48, 49].

Since then, concerns have grown, with once supportive bodies reversing their positions. The Academy of Medical Royal Colleges (AoMRC), the umbrella organisation for the colleges, has called for a review of the role of PAs [50], although a subsequent document dismissed the accumulating evidence giving rise to concern, including the extensive material referenced in this paper, as a “whirlwind of anecdotes [51]. The Royal College of General Practitioners, which had been very supportive, now formally opposes the PA role within general practice [52]. The Royal College of Physicians of Edinburgh has documented a series of concerns and said that “no further expansion of the PA workforce should occur in the UK” [53]. The Royal College of Radiologists has “no plans to bring PAs into the College, and we do not anticipate a significant expansion of the role within our specialities” [54]. The Royal College of Anaesthetists has called for a pause in recruitment of anaesthesia associates [45] and called for much closer supervision [55]. The Royal College of Surgeons has noted how the expansion of PAs could undermine the roles of surgical care practitioners, surgical first assistants and advanced nurse practitioners [56]. The Royal College of Emergency Medicine also “does not currently support the expansion of the Physician Associate workforce in Emergency Medicine” [57]. Few voices are being heard in favour of an expanded role of PAs. Those organisations promoting this role have largely been silent, simply restating the legal situation. The General Medical Council refused, for many months, to release the results of its consultation on PAs despite many calls to do so. The Faculty of Physician Associates has had a change in leadership and closed on 31st December 2024 [58]. Most of the support for an expanded role is found on social media. 

The situation, at the time of writing, is in flux. The GMC is establishing a statutory register and, in response to concerns, has conceded that the format for registration numbers of doctors and physician associates should differ. It has also stated that while it will take on the task of registration, it has no plans to fully ‘regulate’ PAs for several years and has not yet said how this ought to happen. It has declined to set a national scope of practice, leaving others to fill the vacuum [59]. It is also taking forward a series of changes that would reduce the duration and rigour of postgraduate medical training [60]. This seems to reflect the view that, as doctors can access the internet on their phones, they no longer need the knowledge they were once expected to have [61]. Meanwhile, employers are reappraising their use of PAs, especially in the light of the BMA ‘s and Royal College of General Practitioners’ guidance, and while empirical evidence is limited, there are increasing accounts on social media and in the professional press suggesting that some are pausing recruitment or narrowing their scope of practice. As noted previously, PAs have been stopped from assuming some of the most concerning roles, such as child protection.

NHS England has not revisited the Long-Term Workforce Plan, including the expansion of the non-medical workforce, but the new Labour health minister, Wes Streeting, appointed after the Conservative defeat in the July 2024 general election, has said that he supports a role for PAs in the NHS but that the concerns voiced by doctors must be taken seriously [62]. He has commissioned a review of the role of PAs, but this will not report until after the legislation comes into force [63]. The gap between the medical profession and the organisations governing and overseeing healthcare has, arguably, never been so wide.

## Moving forward

The NHS does face a medical workforce crisis. However, there is now widespread agreement among health professionals, the public, and many PAs that using PAs in place of doctors is not the way forward. Some of the surveys described earlier have provided insights into what doctors want to make their jobs easier. These include improved IT systems, with many existing ones seen as slow and inefficient, better administrative support, and access to rest areas and food at unsocial hours, things that are commonplace in many other health systems. There are also concerns about how an expansion of PAs could make things worse. For example, as they must work under direct consultant supervision they will, in most specialities, only be able to work during standard working hours. In some hospitals, resident doctors must cover all the unsocial hours, with consequences for their family life and training.

This raises the question of what to do with those PAs who are already in post. The immediate priority is to define a scope of practice that takes, as its starting point, their training. There is a broad consensus that this should not include seeing patients on their first appointments, as has been happening. However, beyond that, the situation has been complicated by the refusal of the General Medical Council to support a national agreement, instead leaving it to local employers despite the now extensive evidence that this leads to problems.

Defining such guidance needs careful thought about what health professionals do. The PA concept implicitly sees medicine as a series of tasks to be undertaken, many of which can be done by others. However, that is oversimplistic. It involves reasoning based on a detailed understanding of how the body works, knowledge acquired in the anatomy, physiology, biochemistry, and pathology courses that largely fall outside PA training. This need for reasoning explains why, other than with very specific activities such as image recognition, artificial intelligence has failed to replace doctors [64].

There have now been two attempts to define such a scope of practice. Both the Academy of Medical Royal Colleges (AoMRC) and the (BMA) published documents in early March 2024. The AoMRC document was written at pace to pre-empt the BMA document, which it had received under embargo, and was subsequently endorsed by NHS England, which in turn wrote a letter to hospital chief executive officers and medical directors. The letter, which now can be considered NHS policy, outlined a series of high level principles around the use of PAs and AAs [65]. These included that: PAs and AAs are not substitutes for doctors and should not be included on medical rotas; they should not prescribe; there must be clarity about their role; they must be appropriately supervised. The document from the BMA was more radical. It built on its own survey of over 18,000 doctors, of whom 87% reported that the way PAs currently work sometimes or always poses a risk to patient safety. Rather than leaving decisions on scope to employers, it provided detailed guidance. PAs were considered unable to see undifferentiated patients, make independent initial assessments or set treatment plans and they should always be working under direct supervision of a senior doctor. A similar document is expected from the Royal College of Physicians of London in late 2024.

Beyond this, we can envisage two trajectories. The first, based on the role of assistant médicaux in France, would be to see PAs as they were originally envisaged, as doctors’ assistants, undertaking administrative tasks and simple clinical procedures, such as commencing infusions and undertaking certain investigations. This could be a fulfilling role and could greatly alleviate the pressure on doctors. The second would be to explore mechanisms to encourage and support those who wish to undertake a postgraduate medical degree. A few PAs have now made the transition to medical student. In interviews published in the BMJ they expressed discontent with their PA training, such as “I knew there were gaps in my knowledge, but I wasn’t 100% aware of where they were and how deep” and “I don’t feel as though I necessarily got taught enough at university” [66]. In particular, they felt that the 15 days of training they received in specialities such as paediatrics was inadequate and described the activities being carried out as “crazy”. As one concluded “And I also really feel for medical colleagues, who are feeling devalued and seeing the expansion of this other role that has less training, better working conditions, and better pay”.

The current situation is unfair to PAs, who have been misled about their career prospects but do have much to offer, but someone will have to accept that the status quo is not an option and do something about it.

## Discussion

Our case study is subject to several limitations. Ideally, we would be able to draw on empirical data on the outcomes associated with deployment of PAs. However, those studies that have been undertaken are small (six PAs in one widely quoted study [67]). Crucially, while it may seem intuitive that adding staff would be beneficial, this is not always the case, possibly because the trained staff inappropriately delegate responsibilities [68]. We are also aware of how researchers have sought to obtain data from hospital information systems but have failed, mostly because of data completeness (e.g. NHS Resolution, which handles compensation claims, does not have a code for PAs [69]), but also because of an unwillingness to provide the data. Some has been obtained following Freedom of Information requests but NHS bodies have often sought exemptions from replying [70]. It is also limited by the secrecy surrounding this issue, exemplified by the prolonged refusal by the General Medical Council to release the results of its consultation, as noted above.

Undoubtedly, the health systems in all parts of the United Kingdom are facing a workforce crisis, with general practice especially hard hit. However, our analysis shows that the introduction of PAs is an example of how not to produce a sustainable solution. What lessons can other countries, many of which are facing similar shortages of doctors, learn from this experience [8]?

First, while the creation of new occupational groups is often led from the bottom up, their deliberate introduction into a new landscape ought to have clear objectives. Which of the many problems facing the NHS workforce were PAs meant to address? Was it simply a means to increase the number of health workers? If so, then why not concentrate on measures to stem the loss of skilled health workers already in place? Instead, cuts to medical training budgets, in part to support the training of PAs, are preventing doctors close to the end of their specialisation unable to complete it. In 2020, almost 700 individuals were so affected, almost the same as the number of unfilled consultant posts [71]. And why go for a completely new occupational group when existing professions, such as nurses and pharmacists, are already expanding their roles, often successfully? There is no obvious answer to the question of what a physician associate brings to a multidisciplinary team that the existing professional groups do not. The failure to answer this question has contributed to the confusion about scope of practice, a situation not helped by the official policy of allowing this to be defined by employers. This has left supervising doctors in an invidious position and PAs prone to exploitation.

Second, our analysis supports the view that safety of patients must be paramount when introducing new policies. In this instance, expanding new types of workers outpaced the coming of regulation and definitions of scope of practice. From the outset, the colleges were particularly supportive and, in some cases, even enthusiastic about physician associates. However, this increasingly became untenable as doctors have expressed increasing concerns.

Third, recognising that any policy involving changes in roles and responsibilities is, by its nature, complex and thus at risk of unintended consequences, it should be tested, for example, using a systems analysis. When this was done in relation to another response to the workforce crisis, medical school expansion, it revealed numerous problems [42]. In this case, there has been little meaningful discussion about the consequences.

Other European countries are exploring the opportunities for staff who can relieve doctors’ workloads although their scope is much more limited than in the UK. In France, for example, *assistants médicaux*, created in 2019, undertake mostly administrative tasks, including record keeping and coordination of appointments with other professionals, maintaining equipment, and basic tasks such as taking temperatures and blood pressure [72]. The nearest equivalent is the emergence of PAs in Ireland, although no regulatory system exists [73]. Given these developments, the UK’s experience provides an opportunity for other countries to learn from and, already, the European Union of General Practitioners/Family Physicians has made clear that PAs should not be seeing undifferentiated patients in primary care [74]. First, there must be a nationally defined scope of practice that can give confidence to patients and the public. In all health systems, new ways of working are essential and should be welcomed. These include transfers of roles and responsibilities from doctors to other professions, such as nurses, to occupations in supportive roles, such as physician and administrative assistants, and to patients and their carers. However, each of these transfers is different. The health professions, such as medicine and nursing, have their own distinct bodies of knowledge and skills. When they are responsible for supervising occupational groups that support them and are accountable for their actions, they must have absolute clarity about their scope of practice.

Second, there is a need for clarity about the problem to be solved. Is it to reduce the work of doctors? It is already clear that if PAs are supervised adequately, it will add substantially to their workload while placing them at risk if things go wrong. Freedman has examined the paradox whereby there are increasing patient contacts in general practice in the United Kingdom but patients face growing difficulty in getting an appointment [75]. He argues eloquently that this can be explained by the loss of continuity of care, with patients often having to see multiple staff, including PAs, before they reach one who can address their needs while, seeing a health professional who knows them reduces the length of appointments and avoidable hospital admissions. Doctors might be helped more by better information technology and administrative support. Or is it to add expertise to multidisciplinary teams, as is often suggested? If so, someone must set out what specific contribution, distinct from doctors, nurses, and others, they bring [76].

Third, change must be based on trust [77]. Unfortunately, this policy coincided with a series of other developments that have left the morale of the medical profession, especially doctors in training, at a very low ebb. Another grievance has been the elision of the role of PAs from being assistants to workforce substitution. This has led to many doctors feeling that they have been deceived.

Finally, there is a need for a long-term perspective. PAs in the UK offered an attractive quick fix to seemingly intractable staffing problems. Managers could justify paying high salaries as the alternative was employing doctors through locum agencies that would take their cut. However, this has inevitable consequences for training the next generation of medical specialists and general practitioners.

In conclusion, new roles have emerged in the health sector throughout history, and those already there have evolved. However, changes have consequences, and the challenge is to seize the opportunities and avoid the mistakes.

Box 1: Methods and reflexivity As two of us (MM, LV) have been involved in the events described, while this is a case study rather than original research, it is important to describe our approach. While we were already aware of most of the relevant material, to ensure completeness, we undertook the following additional measures. First, we searched PubMed using the following search terms: “(((“physician associate*”[Title]) OR (anaesthetic associate[Title])) AND (English[Language])) AND (United Kingdom[Affiliation])”. This search, undertaken on 11th October 2024, yielded 14 records, 2 of which were irrelevant. Second, we reviewed the references cited in a recent systematic review of PAs and advanced nurse practitioners in the UK by Wang and colleagues, although much of the evidence reviewed concerned the latter staff group [17]. Third, we searched Google Scholar using the terms “physician associate” and “United Kingdom” and screened the titles and abstracts of the first 100 records, reading the full text of 18 additional papers that were potentially relevant to our case study (although few were). Fourth, we searched for the term “physician associate” and the titles of the most widely read national newspapers in the UK. Fifth, we systematically searched the web pages of the medical Royal Colleges and related specialist societies, the BMA, and the Faculty of Physician Associates. We did not systematically search for references on social media, although we are aware of many of them, and have not used them in this case study except to the extent that they have drawn our attention to other sources.

Given the involvement by two of us in this issue, it is also important to engage with reflexivity. MM has been a long-term advocate of task-shifting. His doctoral thesis, in the late 1980s, was on the work of hospital doctors out of hours and advocated that many tasks could be undertaken by others [78, 79]. He has also called for greater task-sharing in academic publications and in a major report for the European Commission [6]. However, he became concerned about the role of PAs because of what he saw as the failure of the medical regulator to recognise or address what he perceived as genuine concerns, leading him to question its accountability [80]. LV has conducted research around smaller, rural and remote hospitals for over a decade and seen good examples of where task-shifting has worked to the advantage of patients and organisations. However, a commissioned piece of research for the Nursing and Midwifery Council alerted her to the problems created by the delegation of scope to employers. As she was an officer and Council member of the Royal College of Physicians, she felt an obligation to address this issue where it pertained to PAs, the College being intimately tied up in many of the debates discussed here. GR is an editor of the general practice workforce crisis in Europe special collection for this journal but played no part in the editorial handling of this paper.

## Data Availability

No datasets were generated or analysed during the current study.
